# Heart Rate Variability and Cognitive Function as Potential Endophenotypes in Schizophrenia: A Cross-Sectional Observational Study Using First-Degree Relatives

**DOI:** 10.7759/cureus.103778

**Published:** 2026-02-17

**Authors:** Priyadarsini Samanta, Barsha B Parida, Jigyansa I Pattnaik, Rama Chandra Das, Rashmi Kumari, Vedaant Parekh, Jayanti Mishra, Jyotiranjan Sahoo, Laxman Kumar Senapati

**Affiliations:** 1 Department of Physiology, Kalinga Institute of Medical Sciences, Bhubaneswar, IND; 2 Department of Psychiatry, Kalinga Institute of Medical Sciences, Bhubaneswar, IND; 3 Department of Physiology, All India Institute of Medical Sciences, Bhubaneswar, Bhubaneswar, IND; 4 Department of Community Medicine, Institute of Medical Sciences and SUM Hospital, Bhubaneswar, IND; 5 Department of Anaesthesiology, Kalinga Institute of Medical Sciences, Bhubaneswar, IND

**Keywords:** autonomic function, cognition, first-degree relatives, heart rate variability (hrv), schizophrenia, sympathetic dominance

## Abstract

Background

Heart rate variability (HRV) represents beat-to-beat fluctuations in heart rate arising from the dynamic balance between sympathetic and parasympathetic nervous system activity. Altered HRV reflects autonomic dysregulation and has been reported across several psychiatric disorders, including schizophrenia, where it may contribute to cardiovascular risk and cognitive dysfunction.

Aim

The aim of this study was to compare the time-domain and frequency-domain heart rate variability parameters between patients with schizophrenia and their first-degree relatives and to analyze the correlation between heart rate variability indices and cognitive performance within an endophenotypic framework.

Methods

This healthcare-based cross-sectional observational study was performed at the Kalinga Institute of Medical Sciences in Bhubaneswar, India, from October 2023 to October 2024. Fifteen clinically stable subjects diagnosed with schizophrenia according to the Diagnostic and Statistical Manual of Mental Disorders, Fifth Edition (DSM-5) criteria and fifteen age- and gender-matched first-degree relatives without schizophrenia were included. 5-minute resting HRV was assessed once at a single time point using a standardized three-lead electrocardiogram (ECG) after a 10-minute adaptation period. Time-domain parameters measured overall variability, whereas frequency-domain parameters analyzed sympathetic and parasympathetic modulation. Cognitive functioning was assessed using the Wechsler Abbreviated Scale of Intelligence (WASI). To compare the groups, we used the Mann-Whitney U test, and to look for associations, we used Spearman’s rank correlation.

Results

Time-domain heart rate variability parameters were comparable between patients with schizophrenia and first-degree relatives (p > 0.05). In the frequency domain, the low frequency (LF) to high frequency (HF) ratio (LF/HF) was significantly higher in patients with schizophrenia than in their relatives (median 1.57 vs. 0.79; p = 0.041), indicating relative sympathetic predominance. Absolute LF, HF, and very-low-frequency (VLF) power values did not differ significantly between groups (p > 0.05). Within the schizophrenia group, the LF/HF ratio showed a significant positive correlation with WASI scores (r = 0.701, p = 0.004).

Conclusion

Schizophrenia is associated with altered autonomic regulation characterized by an increased LF/HF ratio, suggesting sympathovagal imbalance, while time-domain HRV measures remain comparable to those of first-degree relatives. The observed association between autonomic modulation and cognitive performance supports the relevance of HRV as a potential physiological marker linked to cognitive functioning in schizophrenia. Further longitudinal and genetic studies are required to clarify its role as a potential endophenotypic trait.

## Introduction

Schizophrenia is a chronic and profound psychiatric disorder with a lifelong risk of approximately 1-2%, leading to substantial impairment in social, occupational, and cognitive functioning [1]. Individuals with schizophrenia also experience a significantly reduced life expectancy, largely attributable to increased cardiovascular morbidity and mortality [2]. One of the biological mechanisms proposed to explain this excess cardiovascular risk is dysfunction of the autonomic nervous system, which plays a central role in regulating cardiac activity [3,4].

Heart rate is not constant; instead, it varies continuously in response to physiological and environmental demands such as respiration, physical activity, emotional stress, metabolic needs, and circadian rhythms. HRV refers to these beat-to-beat fluctuations in heart rate and provides a non-invasive quantitative measure of autonomic nervous system regulation [5]. Higher HRV reflects greater parasympathetic (vagal) modulation and autonomic flexibility, indicating an efficient ability to adapt to stressors. In contrast, reduced HRV signifies diminished parasympathetic activity and autonomic rigidity and has been associated with increased cardiovascular risk, impaired stress regulation, and adverse health outcomes [6-8].

The neural regulation of HRV is mediated through the central autonomic network, which integrates signals from the prefrontal cortex, limbic structures, and brainstem nuclei to modulate cardiac function via the sinoatrial node [9]. HRV can be quantified using complementary analytical approaches. Time-domain measures describe the overall magnitude of RR-interval variability using statistical indices such as the standard deviation of normal-to-normal intervals (SDNN) and the root mean square of successive differences (RMSSD) [10]. Frequency-domain measures break down heart rate signals into spectral components, including LF and HF power, which reflect autonomic modulation of cardiac rhythm [11]. The LF/HF ratio is frequently utilized as an indicator of sympathovagal equilibrium [12,13].

Reduced HRV has been consistently observed across various psychiatric disorders when compared to healthy populations [5]. Importantly, this reduction is observed across multiple diagnostic categories, including schizophrenia, mood disorders, anxiety disorders, and post-traumatic stress disorder, suggesting that autonomic dysregulation represents a transdiagnostic physiological vulnerability rather than a disorder-specific abnormality [6-8].

Despite extensive research, findings regarding specific HRV components in schizophrenia remain inconsistent, particularly when comparing time-domain and frequency-domain measures [14,15]. While some studies report generalized reductions in HRV, others demonstrate selective alterations in spectral indices without significant changes in absolute power measures. These inconsistencies may be attributed to several methodological and biological factors, including age-related autonomic changes, gender differences, illness duration, physical fitness, and physiological aging processes [16,17]. Through standardized analysis of beat-to-beat cardiac variability, HRV provides an objective method to quantify the cumulative influence of these factors [10].

Beyond cardiovascular regulation, HRV has been increasingly linked to cognitive functioning. Models of neurovisceral integration propose that prefrontal cortical regions involved in executive function, attention, and working memory are closely interconnected with limbic and brainstem circuits that regulate autonomic output [8]. Altered autonomic regulation may therefore reflect impaired top-down cognitive control. In schizophrenia, where cognitive deficits represent a core and enduring feature of the illness, autonomic dysregulation may parallel variability in cognitive performance [18,19].

Only a few studies have directly examined the relationship between HRV and cognitive functioning in schizophrenia using standardized cognitive assessments. The WASI scale is a validated and reliable instrument for assessing general intellectual ability and cognitive performance in clinical populations [20]. Exploring autonomic-cognitive associations using such standardized tools may provide insight into shared neurobiological mechanisms.

The concept of an endophenotype refers to a measurable, heritable biological trait that lies along the pathway between genetic vulnerability and clinical expression of psychiatric illness and is observable in unaffected relatives at higher rates than in the general population [21,22]. In schizophrenia research, multiple candidate endophenotypes have been proposed across neurocognitive and neurobiological domains, reflecting intermediate vulnerability markers rather than overt symptoms [23]. Within this framework, altered autonomic regulations indexed by heart rate variability (HRV) have been suggested as a potential physiological endophenotype, with meta-analytic evidence demonstrating reduced vagal activity in patients and similar alterations in first-degree relatives [24].

Accordingly, this cross-sectional observational study was designed primarily to compare time-domain and frequency-domain HRV parameters between patients with schizophrenia and their first-degree relatives. The secondary objective was to investigate the correlation between HRV indices and cognitive performance in patients with schizophrenia.

## Materials and methods

Study design

This study was a hospital-based, cross-sectional observational study with an analytical patient-relative comparison, carried out between October 2023 and October 2024 at the Kalinga Institute of Medical Sciences (KIMS), Bhubaneswar, India. Participants were enrolled after obtaining Institutional Ethics Committee approval (September 2023). All assessments were performed between 9 and 11 a.m. to avoid diurnal variation.

Participants

The study included 30 participants, comprising 15 patients with schizophrenia (SCZ group) and 15 first-degree relatives without schizophrenia (REL group). The SCZ group consisted of subjects aged 18 to 60 having a verified diagnosis of schizophrenia made in accordance with the DSM-5 by a consultant psychiatrist trained in the application of DSM-5 criteria [25]. To reduce the impact of acute symptom exacerbation on autonomic measures, only clinically stable patients with a minimum illness duration of one year were included. Patients with comorbid psychiatric disorders, neurological illness, cardiovascular disease, diabetes mellitus, or substance use disorders were excluded, as these conditions may independently influence HRV.

REL group participants were age (±2 years) and gender-matched first-degree relatives of the patients. They had no personal history of psychiatric illness and were screened to exclude neurological disorders, chronic medical conditions, or use of medications or substances known to affect autonomic or cognitive function. First-degree relatives were selected to minimize genetic and environmental heterogeneity and to facilitate evaluation of HRV within an endophenotypic framework.

Sample size calculation

The sample size was calculated based on the disparity in resting-state SDNN reported by Liu et al. [26], who demonstrated a mean SDNN of 40.24 ± 19.8 ms in patients with schizophrenia and 23.20 ± 12.88 ms in healthy subjects, corresponding to an expected mean difference of approximately 17 ms. Utilizing this effect size, a two-tailed alpha error of 0.05, 80% power, and a group allocation ratio of 1:1, the necessary sample size was computed employing the conventional formula for the comparison of two independent means:

n = 2 × (Zα/2 + Zβ)² × σ² / δ²,

where n is the number of participants needed in each group, Zα/2 is the value for a two-tailed alpha of 0.05 (1.96), Zβ is the value for 80% power (0.84), σ is the combined standard deviation, and δ is the expected difference between the averages of the two groups. According to these assumptions, the calculated sample size was 16 participants per group. Sample size estimation was obtained using IBM Statistical Package for the Social Sciences (SPSS), version 27 [27]. Considering feasibility constraints and the exploratory nature of this cross-sectional observational study, 15 participants were ultimately recruited in each group.

Ethical considerations

All participants were informed in detail about the study objectives, procedures, and potential risks. Informed consent in writing was secured before enrollment. The Institutional Ethics Committee at KIMS authorized the study (KIIT/KIMS/IEC/1381/2023 dated 18/09/2023), and all methods adhered to ethical guidelines for research involving human participants.

Data collection

Sociodemographic and relevant clinical information were collected using a structured, investigator-administered proforma that included age, sex, education, marital status, illness duration, medication status, and relevant medical history. All assessments were completed during a single study visit for both groups.

Heart rate variability assessment

Participants were instructed to refrain from caffeine and tobacco use for at least six hours prior to assessment to minimize autonomic confounding. After a 10-minute adaptation period, resting HRV was recorded during a continuous 5-minute ECG acquisition with participants in a relaxed supine position to ensure physiological stabilization. ECG signals were obtained using a PowerLab-26T data acquisition system with LabChart 8 software and the HRV Module (version 2.0; ADInstruments, Australia). RR interval data were visually inspected, and ectopic or abnormal beats were removed to generate normal-to-normal (NN) interval series using the module’s artifact detection and correction tools.

HRV analysis was performed across time-domain, frequency-domain, and non-linear domains. Time-domain measures included RR interval, SDRR, SDNN, RMSSD, pRR50, and CVRR. Non-linear HRV indices included the standard deviation of short-term variability (SD1) and the standard deviation of long-term variability (SD2), calculated from the NN interval series to characterize short- and long-term autonomic variability. Frequency-domain measures were derived from the NN interval series using the Lomb periodogram nonparametric spectral method implemented in the HRV module, which estimates spectral power directly from unevenly sampled tachograms. HRV spectral power was calculated across standard frequency bands, including low-frequency (LF: 0.04-0.15 Hz) and high-frequency (HF: 0.15-0.40 Hz) components, and expressed in absolute units. Only recordings with sufficient artifact-free NN intervals were included in the final analysis. The following parameters were calculated and described in Table 1.

**Table 1:** Description of parameters/metrics of HRV calculated in the study

Metrics	Description
Time domain	
RR	RR interval in ms
SDRR	Standard deviation of RR interval in ms
SDNN	Standard deviations of normal-to-normal interval for each 5 min in ms
RmSSD	Root mean square of successive RR interval differences in ms
pRR50	Percentage of successive RR intervals that differ by more than 50 ms
CVRR	Coefficient of Variation of RR intervals in ms
SD rate	Standard deviation of the heart rate in beats per minute
SDSD	Standard deviation of successive differences in ms
Frequency domain	
Total power	Overall variance in heart rate patterns including all frequencies in ms^2^
VLF power	Absolute power of the very-low-frequency band (0.0033–0.04 Hz) in ms^2^
LF power	Absolute power of the low-frequency band (0.04–0.15 Hz) in ms^2^
HF power	Absolute power of the high-frequency band (0.15–0.4 Hz) in ms^2^
LF/HF	Ratio of LF-to-HF power in %
Nonlinear	
SD1	Standard deviation of short-term variability in ms
SD2	Standard deviation of long-term variability in ms

Cognitive assessment

Cognitive assessment using the WASI was conducted by a trained professional following standard administration procedures, a validated and reliable measure of general intellectual ability suitable for clinical populations [20]. The Full-Scale IQ (FSIQ) score from the WASI was used for analysis. The assessment was administered on the same day as the HRV recording in a quiet setting, following standard administration and scoring procedures.

Outcomes

The primary outcomes were between-group differences in time-domain and frequency-domain HRV parameters. Frequency-domain measures, including the LF/HF ratio, were analyzed exploratorily. The secondary outcome was the association between HRV indices and cognitive performance, as measured by WASI scores, within the schizophrenia group.

Statistical analysis

Variables were put into Microsoft Excel and analyzed using IBM Corp. Released 2021. IBM SPSS Statistics for Windows, Version 27. Armonk, NY: IBM Corp. [27]. Categorical variables were summarized as frequencies and percentages. Continuous variables were represented as median and interquartile range (IQR) because they did not follow a normal distribution, as determined by the Shapiro-Wilk test. Between-group comparisons of continuous variables were performed using the Mann-Whitney U test. Associations between HRV parameters and cognitive performance within the schizophrenia group were examined using Spearman’s rank correlation coefficient. A p-value less than 0.05 was deemed statistically noteworthy.

## Results

A total of 30 participants were included in the analysis, comprising 15 patients in the schizophrenia group (SCZ group) and 15 participants in the first-degree relative group (REL group).

Sociodemographic characteristics

The SCZ and REL groups were equivalent in terms of age and gender distribution, with no statistically significant variations noted (Table 2).

**Table 2:** Sociodemographic characteristics of the study population Values are expressed as median (interquartile range) or number (percentage). Age was compared using the Mann–Whitney U test. Sex distribution was compared using Fisher’s exact test.

Variable	SCZ group (n = 15)	REL group (n = 15)	Test statistic	p-value
Age (years), median (IQR)	35.0 (31.0–50.0)	39.0 (34.0–47.0)	U = 126.5	0.567
Sex, n (%)				
• Male	10 (66.7)	10 (66.7)	—	1.00
• Female	5 (33.3)	5 (33.3)	—	—

Time-domain HRV

Table 3 summarizes the time-domain HRV parameters of the SCZ and REL groups. Median heart rate (beats per minute) was identical between the two groups, with no statistically significant distinctions (p > 0.05). The median values of SDNN, RMSSD, pRR50, CVRR, and other time-domain indices were lower in the SCZ group than in the REL group. However, none of these differences were statistically noteworthy (all p > 0.05). These results show that the SCZ and REL groups had similar levels of overall beat-to-beat variability and parasympathetic modulation, as measured by time-domain HRV measures.

**Table 3:** Comparison of time domain HRV parameters between the SCZ group and the REL group SDNN: Standard deviations of normal-to-normal, RMSSD: Root mean square of successive RR interval differences, CVRR: Coefficient of Variation of RR intervals, SD: Standard deviation, SDSD: Standard deviation of successive differences

Variable	SCZ group, median (IQR)	REL group, median (IQR)	U value	p-value
RR interval (ms)	682.7 (641.9–796.7)	782.8 (686.1–809.4)	142.0	0.233
SDNN (ms)	25.5 (15.6–43.4)	32.5 (18.6–39.1)	138.0	0.305
RMSSD (ms)	15.8 (5.9–36.3)	24.1 (16.1–24.1)	136.0	0.345
pRR50 (%)	0.18 (0–13.7)	2.2 (0.44–12.3)	136.0	0.345
CVRR	0.03 (0.02–0.05)	0.04 (0.02–0.05)	127.0	0.567
Heart rate (bpm)	87.9 (75.4–93.5)	75.1 (74.2–84.4)	67.0	0.061
SD (bpm)	2.72 (1.94–3.97)	3.18 (2.33–4.15)	123.0	0.683
SDSD (ms)	15.9 (5.86–36.4)	24.1 (16.1–32.1)	136.0	0.345

Frequency-domain HRV

Frequency-domain HRV parameters are presented in Table 4. Absolute power values for total power, very-low-frequency (VLF), low-frequency (LF), and high-frequency (HF) bands did not differ significantly between the SCZ and REL groups (all p > 0.05). In contrast, the LF/HF ratio was significantly higher in the SCZ group than in the REL group (median [IQR]: 1.57 [1.05-2.62] vs. 0.79 [0.43-1.52]; p = 0.041), indicating a relative alteration in sympathovagal balance in the SCZ group.

**Table 4:** Comparison of frequency domain HRV parameters between the SCZ group and the REL group VLF: Very-low-frequency, LF: Low frequency, HF: High frequency Values are expressed as median (interquartile range). Between-group comparisons were performed using the Mann–Whitney U test. A p-value < 0.05 was considered statistically significant.

Variable	SCZ group, median (IQR)	REL group, median (IQR)	U value	p-value
Total power (ms²)	442.6 (239.0–1138.0)	880.1 (128.4–506.2)	133.0	0.412
VLF power (ms²)	218.3 (86.4–599.4)	229.0 (128.4–506.2)	122.0	0.713
LF power (ms²)	127.1 (60.4–282.6)	235.6 (57.8–376.2)	128.0	0.539
HF power (ms²)	89.0 (27.2–262.3)	148.1 (97.4–453.8)	151.0	0.116
LF/HF ratio	1.57 (1.05–2.62)	0.79 (0.43–1.52)	63.0	0.041

Non-linear HRV and cognitive performance

**Figure 1:**
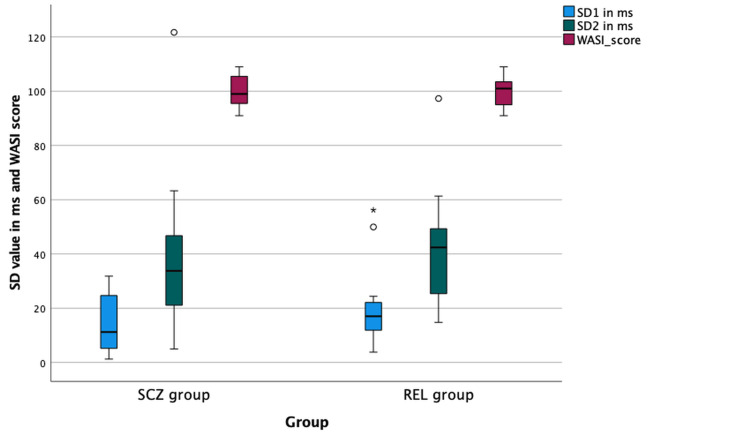
Box plot showing comparison of non-linear HRV indices (SD1, SD2) and WASI scores between the SCZ group and the REL group SD1: Standard deviation of short-term variability, SD2: Standard deviation of long-term variability, WASI: Wechsler Abbreviated Scale of Intelligence, SCZ: Schizophrenia, REL: Relative group The box represents the interquartile range, the central line denotes the median, and the whiskers indicate data dispersion.

Group comparisons of nonlinear HRV indices (SD1 and SD2) and WASI scores are illustrated in Figure 1. Median values of SD1, SD2, and WASI scores did not differ significantly between the SCZ and REL groups (all p > 0.05). SD1 reflects short-term beat-to-beat variability predominantly influenced by parasympathetic modulation, whereas SD2 represents long-term variability and overall autonomic regulation. Figure 1 visually demonstrates the comparability of nonlinear autonomic dynamics and global cognitive performance between the two groups.

Association between HRV and cognitive function

**Figure 2:**
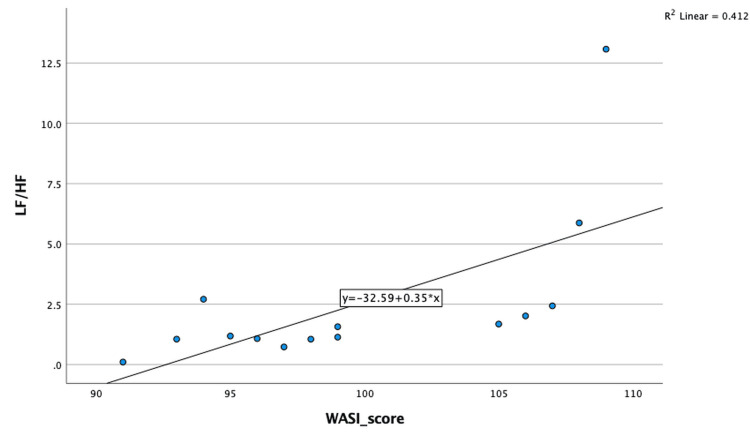
Scatter plot depicting the correlation between LF/HF ratio and WASI scores in the SCZ group LF: Low frequency, HF: High frequency, WASI: Wechsler Abbreviated Scale of Intelligence, SCZ: schizophrenia

Within the SCZ group, Spearman correlation analysis demonstrated a significant positive association between the LF/HF ratio and WASI scores (Spearman’s ρ = 0.701, p = 0.004), as shown in Figure 2. No significant correlations were observed between WASI scores and time-domain or non-linear HRV parameters in the SCZ group. Additionally, no significant associations between HRV parameters and cognitive performance were identified in the REL group.

## Discussion

Overview of the study findings

This hospital-based cross-sectional observational study examined HRV parameters and cognitive performance in patients with schizophrenia and their first-degree relatives to explore autonomic regulation within a potential endophenotypic framework. The principal findings were that most time-domain and non-linear HRV parameters were comparable between groups, whereas the LF/HF ratio was significantly higher in the SCZ group. Additionally, within the SCZ group, a significant positive association was observed between the LF/HF ratio and cognitive performance, as measured using the WASI scale. A key strength of this study is the inclusion of first-degree relatives as a comparator group, enabling evaluation of autonomic characteristics within an endophenotypic framework and providing insight into familial vulnerability.

Autonomic regulation in schizophrenia

Time-domain HRV indices such as SDNN and RMSSD, which reflect overall variability and parasympathetic modulation, did not differ significantly between patients with schizophrenia and their first-degree relatives. Previous studies evaluating HRV in schizophrenia have reported heterogeneous findings, with several demonstrating reduced parasympathetic activity and overall HRV, while others have reported relatively preserved autonomic modulation, particularly in clinically stable patients [14,15]. Such variability has been attributed to differences in age, illness duration, antipsychotic exposure, symptom severity, and methodological factors across studies [16,17]. The present findings suggest that, in clinically stable patients, resting-state time-domain HRV measures may not differ markedly from those observed in biologically related relatives.

In contrast, the significantly elevated LF/HF ratio observed in the SCZ group indicates a relative alteration in sympathovagal balance. Importantly, this occurred despite comparable absolute low-frequency and high-frequency power values between groups. Alterations in the LF/HF ratio have been widely reported in schizophrenia and are often interpreted as reflecting autonomic imbalance rather than absolute reductions in autonomic activity [18,19]. Systematic reviews have further highlighted consistent HRV alterations across psychiatric disorders, including schizophrenia, while emphasizing heterogeneity in specific HRV indices depending on study design and patient characteristics [28].

Non-linear HRV and group comparability

Non-linear HRV indices, including SD1 and SD2, which represent short-term and long-term autonomic variability, respectively, did not differ significantly between the SCZ and REL groups. Non-linear measures capture complex autonomic dynamics and have been proposed as sensitive markers of autonomic dysregulation; however, previous studies in schizophrenia have yielded mixed results, particularly in stable or chronic populations [19]. The similarity of nonlinear HRV indices between patients and their first-degree relatives in the present study may reflect shared genetic or environmental influences on autonomic regulation, supporting the possibility of trait-like autonomic characteristics within families affected by schizophrenia.

HRV and cognitive function

A key finding of this study was the significant positive association between the LF/HF ratio and cognitive performance within the SCZ group. According to the neurovisceral integration model, autonomic regulation is closely linked to prefrontal cortical networks involved in executive function, attention, and cognitive control [8]. In schizophrenia, where cognitive impairment is a core and enduring feature, autonomic imbalance may reflect disrupted top-down regulatory mechanisms.

Previous investigations have demonstrated associations between altered HRV and functional, clinical, and cognitive outcomes in schizophrenia and related psychotic disorders [18,29]. Kimhy et al. reported that autonomic dysregulation was associated with functional impairment in schizophrenia, underscoring HRV as a physiological marker of central regulatory dysfunction [29]. The present findings extend this literature by demonstrating an association between sympathovagal balance and global cognitive performance using a standardized cognitive assessment, though this relationship should be interpreted cautiously given the exploratory nature of the analysis.

HRV as a potential endophenotypic marker

Within an endophenotypic framework, biological traits that are shared between patients and their unaffected relatives are interpreted as markers of familial vulnerability rather than illness-specific changes [21,23]. In the present study, the similarity of most time-domain and nonlinear HRV parameters between the schizophrenia and relative groups suggests that aspects of autonomic regulation may reflect trait-like or familial characteristics. These findings should be interpreted as preliminary and exploratory rather than confirmation of a definitive endophenotypic marker. This interpretation is consistent with prior work proposing heart rate variability as a potential physiological endophenotype in schizophrenia, particularly given evidence of comparable autonomic alterations in patients and their first-degree relatives [24].

Clinical and research implications

Autonomic dysregulation has been implicated in the increased cardiovascular morbidity and mortality observed in individuals with schizophrenia [2,3]. The presence of altered sympathovagal balance, even among clinically stable patients, highlights the importance of cardiovascular risk monitoring in this population. Furthermore, the observed association between HRV and cognitive performance suggests that autonomic markers may offer insight into broader neurobiological mechanisms underlying cognitive dysfunction in schizophrenia, with potential implications for early identification and targeted interventions.

Limitations

Multiple limitations should be acknowledged. The modest sample size limits statistical power, increases uncertainty around effect estimates, and reduces generalizability. Accordingly, the findings should be interpreted as exploratory and hypothesis-generating rather than definitive. The potential influence of psychotropic medication on HRV was not analyzed separately and may have affected autonomic measures. The cross-sectional approach prevents causal inferences regarding the relationship between autonomic regulation and cognitive performance. Additionally, HRV assessment was limited to resting-state recordings; task-based or stress-induced autonomic paradigms may have revealed additional group differences.

Future directions

Future studies should employ larger, longitudinal designs to better clarify the temporal relationship between autonomic dysregulation and cognitive impairment in schizophrenia. Familial and genetic studies may further elucidate the role of HRV as an endophenotypic marker, while interventional studies targeting autonomic regulation could explore potential therapeutic benefits.

## Conclusions

In this cross-sectional observational study, resting-state heart rate variability parameters were largely comparable between patients with schizophrenia and their first-degree relatives, with the exception of a significantly higher LF/HF ratio in patients, indicating a relative alteration in sympathovagal balance. The observed association between the LF/HF ratio and cognitive performance within the schizophrenia group suggests a potential link between autonomic regulation and cognitive functioning in this population. The similarity of most time-domain and nonlinear HRV measures between patients and relatives supports the possibility that certain autonomic features may reflect shared, trait-like characteristics rather than illness-specific changes alone. Given the exploratory nature and modest sample size of the study, these findings should be interpreted as preliminary and hypothesis-generating. Larger, longitudinal studies incorporating task-based autonomic assessments are warranted to further clarify the role of heart rate variability as a potential endophenotypic marker and its clinical relevance in schizophrenia.
